# Identification of novel Kirrel3 gene splice variants in adult human skeletal muscle

**DOI:** 10.1186/s12899-014-0011-3

**Published:** 2014-12-09

**Authors:** Peter Joseph Durcan, Johannes D Conradie, Mari Van deVyver, Kathryn Helen Myburgh

**Affiliations:** Department of Physiological Science, Stellenbosch University, Private Bag X1 Matieland, 7602 Stellenbosch, South Africa; Division of Endocrinology, Department of Medicine, Stellenbosch University, Tygerberg, South Africa

**Keywords:** Kirrel3, Human, Myogenesis, Biopsy, Drosophila

## Abstract

**Background:**

Multiple cell types including trophoblasts, osteoclasts and myoblasts require somatic cell fusion events as part of their physiological functions. In Drosophila Melanogaster the paralogus type 1 transmembrane receptors and members of the immunoglobulin superfamily Kin of Irre (Kirre) and roughest (Rst) regulate myoblast fusion during embryonic development. Present within the human genome are three homologs to Kirre termed Kin of Irre like (Kirrel) 1, 2 and 3. Currently it is unknown if Kirrel3 is expressed in adult human skeletal muscle.

**Results:**

We investigated (using PCR and Western blot) Kirrel3 in adult human skeletal muscle samples taken at rest and after mild exercise induced muscle damage. Kirrel3 mRNA expression was verified by sequencing and protein presence via blotting with 2 different anti-Kirrel3 protein antibodies. Evidence for three alternatively spliced Kirrel3 mRNA transcripts in adult human skeletal muscle was obtained. Kirrel3 mRNA in adult human skeletal muscle was detected at low or moderate levels, or not at all. This sporadic expression suggests that Kirrel3 is expressed in a pulsatile manner. Several anti Kirrel3 immunoreactive proteins were detected in all adult human skeletal muscle samples analysed and results suggest the presence of different isoforms or posttranslational modification, or both.

**Conclusion:**

The results presented here demonstrate for the first time that there are at least 3 splice variants of Kirrel3 expressed in adult human skeletal muscle, two of which have never previously been identified in human muscle. Importantly, mRNA of all splice variants was not always present, a finding with potential physiological relevance. These initial discoveries highlight the need for more molecular and functional studies to understand the role of Kirrel3 in human skeletal muscle.

## Background

Somatic cell fusion events are critical for development of multicellular eukaryotic organisms. Postnatal growth and repair also frequently rely on somatic cell fusion events, especially in tissues such as skeletal muscle that contain multinucleated cells. However, the genes and molecular mechanisms underpinning this fundamental cellular process in humans are largely unknown. Multiple cell types including trophoblasts, osteoclasts and skeletal muscle require somatic cell fusion events in order to perform their physiological functions [1–3]. The occurrence of cell fusion events in skeletal muscle provides a potential mechanism for the introduction of exogenous DNA to cure genetic myopathies, while increasing evidence is emerging that fusion of cancer cells and bone marrow derived cells may enable and promote cancer metastasis [4–7]. Therefore, an improved understanding of how somatic cells fuse will likely have diverse clinical benefits.

In humans, a single skeletal muscle fibre can contain thousands of nuclei [8]. Each nucleus present in the muscle fibre arises from asynchronous cell fusion events. Post birth, skeletal muscle is a very adaptable tissue that is capable of repairing itself in response to injury such as that which accrues from damage-inducing exercise bouts [9,10]. The regeneration capacity of skeletal muscle is largely due to the presence of stem cell-like progenitor cells, termed satellite cells, that reside between the basal lamina and plasma membrane [11]. Satellite cells are typically found in an inactive state, however, in response to muscle injury they proliferate and also fuse (either together or with damaged fibres) in order to facilitate muscle repair [12–14]. The large amount of fusion events that occur during development, growth and repair of skeletal muscle make it a suitable tissue for studies aiming to identify the genes and mechanisms that underpin the somatic cell fusion process.

Research findings from Drosophila have highlighted that key events in the muscle cell fusion process are cell-cell attraction, adhesion and subsequent actin nucleation at sites of cell-cell adhesion, the latter enabling membrane fusion [15–17]. A wide variety of genes impact on the muscle cell fusion process ranging from actin nucleation factors, such as those found in the actin related protein 2/3 complex [18], to type 1 transmembrane receptors [19,20]. Of particular interest to this research paper has been the discovery of two paralogus type 1 transmembrane receptors and members of the immunoglobulin (Ig) superfamily Kirre [21] and Rst [22]. Loss of both Kirre and Rst from the Drosophila genome results in muscle cell fusion inhibition [19].

Present within the human genome are three Kirre homologs termed Kin of Irre like (Kirrel) 1 ,2 and 3 [23]. This family of genes have also been referred to in the literature as the Neph family, Neph1 (Kirrel1), Neph2 (Kirrel3), and Neph3 (Kirrel2)[23,24]. Kirrel3 has been implicated in diverse functions including pontine nuclei formation in the developing brain [25] male-male aggressive behaviour [26] and inhibition of hematopoietic stem cell differentiation [27]. Notably, no detailed investigation has been performed on any of the Kirrel family members and their presence in human skeletal muscle. Of particular interest to this research paper is the Kirrel3 gene as murine kirrel3 has been reported to be present in the kidney [28], brain [25] and also in cultured stromal cells [27], yet, results from mRNA analysis of murine skeletal muscle suggest that Kirrel3 is absent [27]. However, a different study reported Kirrel3 immunoreactivity in lysates obtained from mouse skeletal muscle [28]. These conflicting reports on Kirrel3 in mammalian skeletal muscle merit further investigation.

Our primary aim was to assess whether Kirrel3 is present in uninjured and regenerating human skeletal muscle following mild damage-inducing exercises such as plyometric jumping and downhill running. Presence of Kirrel3 in the afore mentioned samples would raise questions about its function in skeletal muscle. Currently nothing is known about Kirrel3 in this tissue, or if it is indeed present in adult human skeletal muscle. While its presence alone would not confirm a role in the human muscle cell fusion process, it would provide initial support for further investigation. It is possible that it could mirror the role of Kirre, its Drosophila homolog, in regulating myoblast fusion events in adult human muscle.

Our research findings demonstrate that at least three alternative splice variants of Kirrel3 are present in adult human skeletal muscle. Two of these splice variants have not been previously reported in the published literature. Detection of Kirrel3 mRNA in adult human skeletal muscle using standard PCR was sporadic with occasional detection in uninjured and regenerating skeletal muscle samples. Semi nested PCR increased the detection rate. Such sporadic detection, using standard PCR assessment, demonstrates that in adult human skeletal muscle Kirrel3 mRNA is present at very low levels. At the protein level, Kirrel3 immunoreactive proteins in uninjured and regenerating adult human skeletal muscle were observed. Further work is now required in order to ascertain the function of all Kirrel3 splice variants in human skeletal muscle and to provide more insight into stimuli promoting its expression and its subsequent post-transcriptional regulation.

## Results

**Figure 1:**
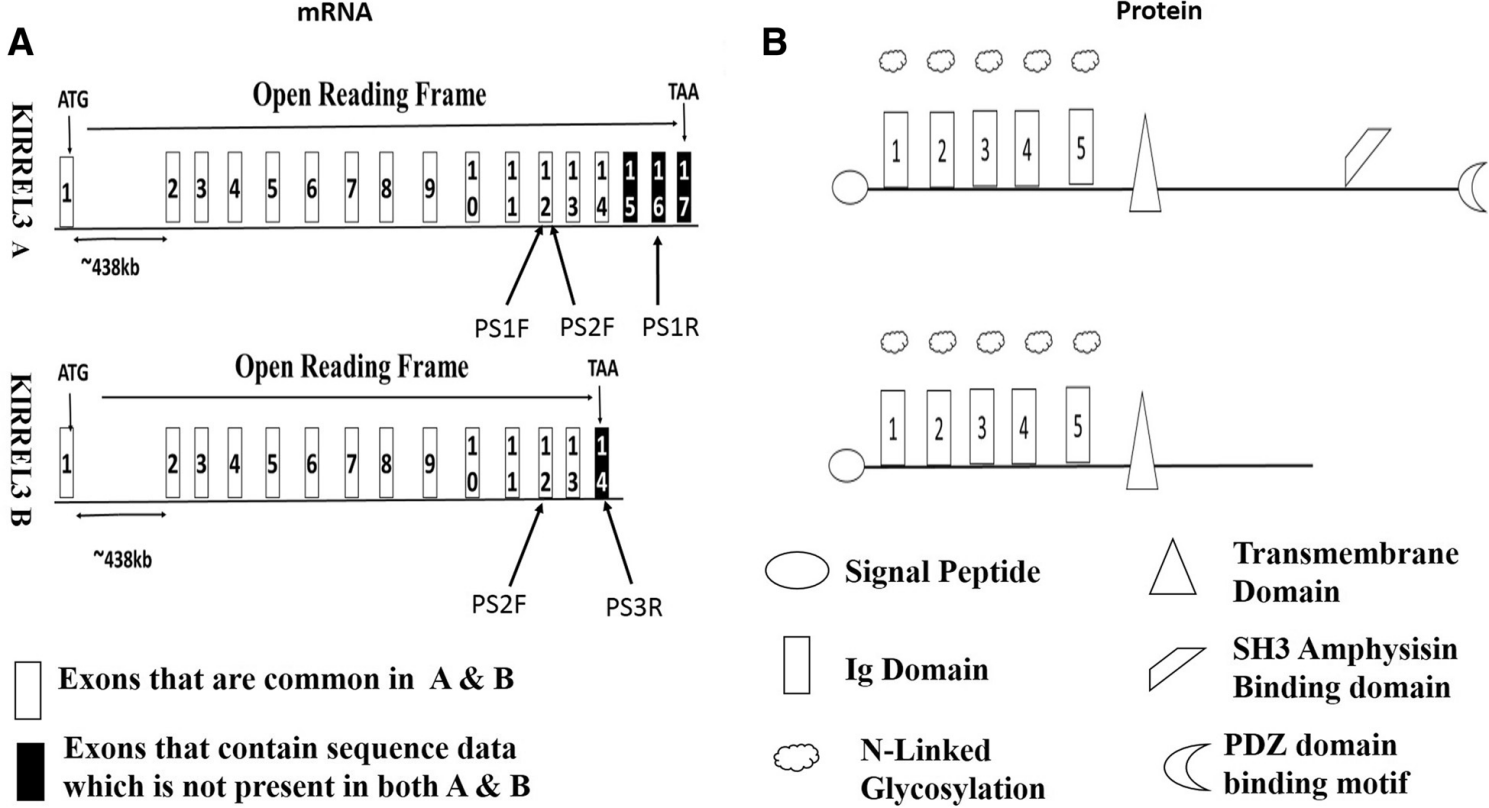
**Schematic of the exon structure of Kirrel3 A and B mRNA transcripts. A** – Schematic of the exon structure of Kirrel3 A and B mRNA transcripts. Start codon is highlighted as ATG and stop codon as TAA. The unusually large first intron is highlighted. The exons to which primers are targeted are also shown. PS1F – Primer set 1 forward primer, PS2F – Primer set 2 forward primer, PS1R – Primer set 1 reverse primer, PS3R – Primer set 3 reverse primer. **B** – Schematic of the predicted protein domains and putative N-linked glycosylation sites of Kirrel3 **A** and **B**.

Analysis of the National Centre for Biotechnology (NCBI) gene database highlighted the presence of two human Kirrel3 reference sequences NM_032531.3 (hereafter referred to as Kirrel3 A) containing 3777 nucleotides and NM_001161707.1 (hereafter referred to as Kirrel3 B) containing 2534 nucleotides. Aligning Kirrel3 A and B mRNA transcripts to the human genome via DNA sequence present in the genomic contig NT_033899.8, demonstrated that Kirrel3 A has 17 exons while B has 14 exons (see Figure 1A for schematic). Of particular note in both transcripts was the very large first intron which spanned approximately 438 kilo bases (KB). Both transcripts are predicted to start their translation initiation in their first exons. The first 2057 nucleotides of Kirrel3 A and B are identical. This region spans exons 1-14 of Kirrel3 A. Subsequently Kirrel3 A has a splice site that is not present in B thus resulting in exon 14 of Kirrel3 B containing an additional 477 nucleotides. The predicted stop codon (TAA) in Kirrel3 B is 107 nucleotides 3′ to the missed splice site. In comparison to Kirrel3 B, Kirrel3 A has an additional 3 exons totalling 1720 nucleotides 3′ to the missed spliced site. The stop codon (TAA) for Kirrel3 A is located in exon its 17^th^ exon 641 nucleotides 3′ to the missed spliced site in B. The 3′ untranslated regions of Kirrel3 A and B are 1079 and 370 nucleotides respectively.

### *In silico* analysis of Kirrel3 protein domains

Kirrel3 A is the larger of the two Kirrel3 proteins with 778 amino acids (AA) while Kirrel3 B has 600 AA. The first 565 AA of Kirrel3 A and B are identical. Domains predicted within this region (see Figure 1B for schematic) are a signal peptide (AA1-21 http://www.cbs.dtu.dk/services/SignalP/), five extracellular Ig domains (AA 49-146, 152-239, 251-332, 336-417, 420-516 http://scansite.mit.edu/) and a transmembrane domain (AA 536-558 http://www.cbs.dtu.dk/services/TMHMM/). Five putative N-linked glycosylation sites (AA 167, 253, 324, 361 & 498 http://www.cbs.dtu.dk/services/NetNGlyc/) are predicted to be present in the extracellular domain of both Kirrel3 proteins. The intracellular domain of Kirrel3 A is predicted to contain an amphysisin SH3 binding domain (AA 759-773 http://scansite.mit.edu/). Also present at its C-terminal end is a Post Synaptic density protein 95, Drosophila discs Large tumour suppressor, zonula occludens (PDZ) binding domain corresponding to the amino acids THV [29]. Kirrel3 B is not predicted to contain either of the afore mentioned intracellular domains.

### Analysis of mRNA

To assess for the presence of Kirrel3 A and B mRNA in adult human skeletal muscle, gene specific primers were designed. We initially investigated Kirrel3 mRNA expression in adult human male skeletal muscle biopsies obtained pre and 4 and 24 hr post a plyometric jumping exercise in order to assess for Kirrel3 in uninjured and mildly injured skeletal muscle. The plyometric jumping protocol used here has previously been demonstrated to induce mild muscle damage [30]. Kirrel3 has previously been reported to be present in the mouse brain [31] and for the current study human astrocytes were used as a positive control for Kirrel3 mRNA expression.

**Figure 2:**
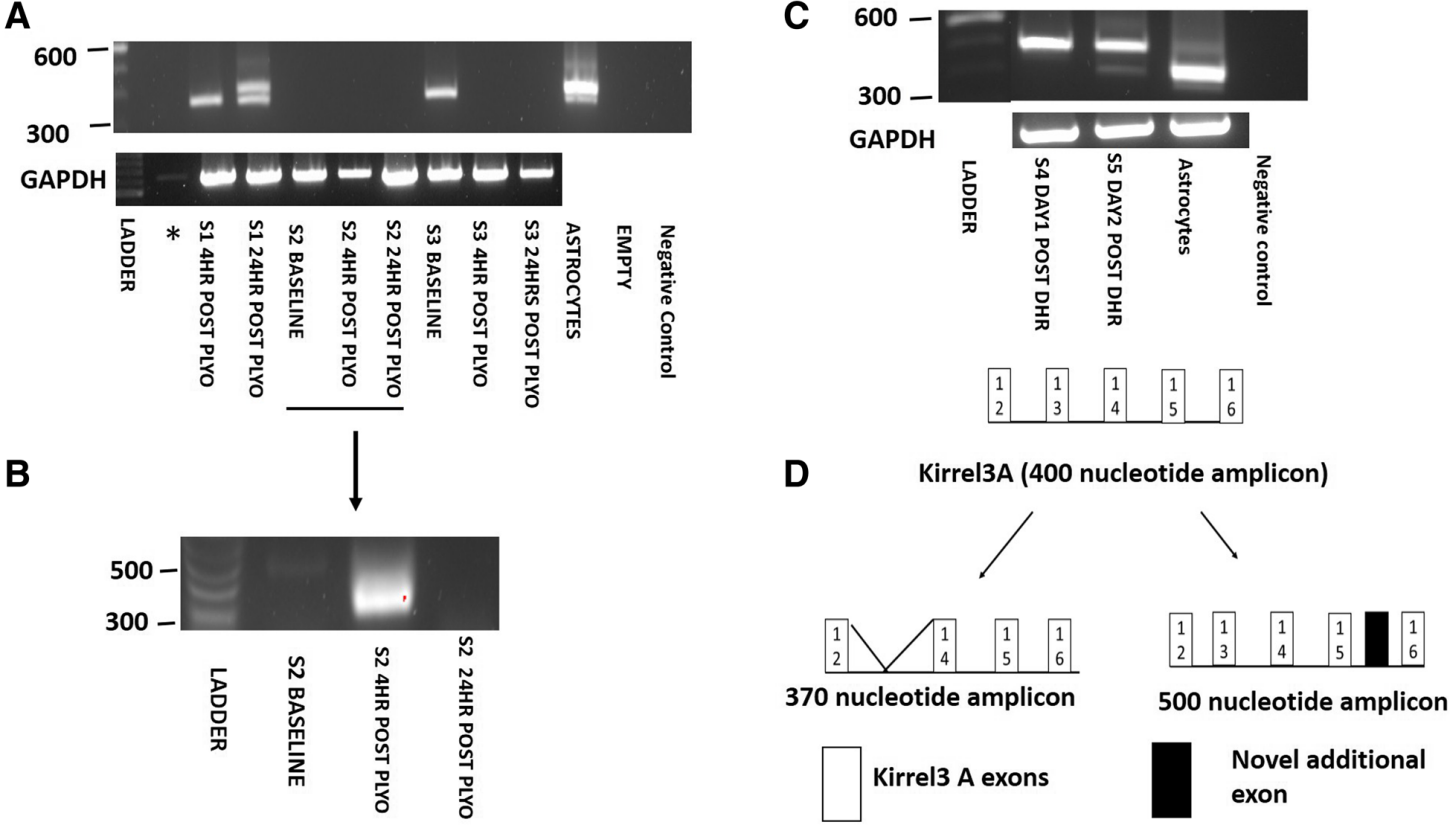
**Detection of Kirrel3 splice variants with primer set 1. A** – PCR amplicons obtained with Kirrel3 primer set 1 to detect Kirrel3 A. mRNA was isolated from adult human skeletal muscle at rest and 4 hr and 24 hr post performance of a plyometric jumping (PLYO) exercise (S = subject) and culture human astrocytes. *Note - the unlabelled lane came from subject 1 baseline, however, its GAPDH level was very low and hence, it has not been commented on in this manuscript. **B** – Results obtained using a nested PCR approach with Kirrel3 primer set 1 and 2 on biopsy samples from subject 2 from Figure A. **C** – Additional PCR amplicon (500 nucleotides) was detected using Kirrel3 Primer set 1 from a subset of adult human skeletal muscle biopsies that were obtained from participants one and two days post a downhill run (DHR) exercise bout. **D** – Schematic of the exon structure of amplicons detected in **A** and **B** after excision of the bands from the gels and nucleotide sequencing.

Primer set 1 was specific to Kirrel3 A with a forward primer targeting exon 12 and a reverse primer targeting exon 16 of Kirrel3 A (see Figure 1A for schematic on primer position). The expected amplicon is 391 nucleotides. Two distinct amplicons migrating at approximately 370 and 400 nucleotides were detected in the biopsy taken from subject 1 at 24 hr post plyometric exercise (Figure 2A). In contrast, only the approximately 370 nucleotide amplicon was detectable in the biopsy sample that was taken at the 4 hr post plyometric exercise time point from the same subject (Figure 2A). No biopsy from Subject 2 presented with either the approximately 370 or 400 nucleotide amplicon. Subject 3 had the approximately 370 nucleotide amplicon present at the baseline time point, but not at 4 hr or 24 hr post plyometric exercise (Figure 2A). The quality of cDNA template in all human skeletal muscle samples (except for subject 1 baseline) was confirmed as satisfactory via assessing for GAPDH mRNA expression (Figure 2A). A semi nested PCR was performed with primer sets 1 and 2 to assess if this approach would yield more consistent Kirrel3 mRNA detection. Primer set 2 contained a forward primer that was targeted towards exon 12 of Kirrel3 A with the reverse primer being the same as in primer set 1 (see Figure 1A for schematic of primer location). Three samples from subject 2 were negative for all amplicons in the first analysis. However, in one of these an amplicon of approximately 370 nucleotides was detected at the 4 hr time point using the semi-nested PCR (Figure 2B). In human astrocytes two amplicons were observed with primer set 1 at approximately 370 and 400 nucleotides (Figure 2A) thus matching those observed in human skeletal muscle. A much fainter amplicon was also detected in astrocytes at approximately 500 nucleotides (Figure 2A). We also analysed mRNA extracted from biopsies obtained from a previous study by our research group that investigated the effects of downhill running on skeletal muscle [32]. In some of these samples, an amplicon was detected that matched of the approximate 500 nucleotide amplicon observed in astrocytes (Figure 2C).

All three differently sized amplicons detected in adult human skeletal muscle samples were excised from the gel and sequenced. Aligning the sequence data obtained from the approximately 370, 400 and 500 nucleotide sized amplicons to the human genome via the Basic Local Alignment Search Tool (BLAST available at http://blast.ncbi.nlm.nih.gov/Blast.cgi) demonstrated that they were all variants of Kirrel3 (see Figure 2D for schematic of exon structure and Table 1 for raw sequence data). The approximately 400 nucleotide amplicon corresponded to a known sequence present in Kirrel3 A. The 37 nucleotide containing exon 13 of Kirrel3 A was spliced out of the approximately 370 nucleotide amplicon. Analysis of the approximate 500 nucleotide amplicon sequence highlighted the presence of an additional exon of 93 nucleotides between exon 15 and 16 of Kirrel3A. This novel exon is located approximately 3100 nucleotides 3′ to exon 15 of Kirrel3 A and 355 nucleotides 5′ to exon 16 of Kirrel3 A.

**Table 1:** **Sequence data obtained from amplicons with Kirrel3 primer set 1**

370 amplicon nucleotide sequence	TARRCWACTGCTGGATMCCCCGGTCATCATCAGCTGCTTGATGGTGGAGTGCTCCTCACCCTCCCGACCAGAGGCTGGTT
	CCTTGTGGACAATTTCCACTCGGATATCATTTTTGGCTGACACAACACCTTTGAGATTTCTCTGGGAACGGGCACAGCAG
	AACGCCACGATGGTTGCCATAAGGACGAGGAAGGCCACACCAGCTCCTACGGCCACCCCAATGATGACGGCCATCGGCAC
	AGACTCTTGCTCCTTGAGCCGGATGATCTCAGTGTCGGAGCCGAAGCTGTTCCAGGCCGTGCAGTTGTAGATGGTCTGGA
	AGTCGGCAAA
400 amplicon nucleotide sequence	GTRMGTACTGCTGGATTMCCCCGGTCATCATCAGCTGCTTGATGGTGGAGTGCTCCTCACCCTCCCGACCAGAGGCTGGT
	TCCTTGTGGACAATTTCCACTCGGATATCATTTTTGGCTGACACAACACCTTTGAGATTTCTCTGGGAACGGGCACAGCA
	GAACGCCACGATGGTTGCCATAAGGACGAGGAAGGCCACACCAGCTCCTACGGCCACCCCAATGATGACGGCCATCGGCA
	CAGACTCTGGTTCCWKSCCSGSTCCCAACTTCRTTTCCGAACCTTGCTCCTTGARCCSGATGATCTCRKWGWYGGASYCG
	AAGYTGKYACAGGCCGTGCAGTTGTAGATGGTCTGGAAGTCGGCA
500 nucletotide sequence	GGRARWRGTCKCACGCTTCGGCTCCGACACTGAGATCATCCGGCTCAAGGAGCAAGGTTCGGAAATGAAGTCGGGAGCCG
	GGCTGGAAGCAGAGTCTGTGCCGATGGCCGTCATCATTGGGGTGGCCGTAGGAGCTGGTGTGGCCTTCCTCGTCCTTATG
	GCAACCATCGTGGCGTTCTGCTGTGCCCGTTCCCAGAGAAATCTCAAAGGTGTTGTGTCAGCCAAAAATGATATCCGAGT
	GGAAATTGTCCACAAGGAACCAGCCTCTGGTCGGGAGGGTGAGGAGCACTCCACCATCAAGCAGCTGATGCAGAGCAACT
	GGCCGGCATTTTACAATAAACGTTCAGTCAATGGAATTGAATCAKCCATGGGAGATCTCTGGTGATGCCCTCGCCCACRC
	GAGATGGACCGGGGTGAATTCCAGCAAGACTCASTCCTGAAACARCTGGAGGTCCTCAAAARACACTYCCTTTYYCCCCC
	CCYCCTTTAMCCCCCTTTTCTCCTCTTTTTTTTCTTCTTTT

Amplicons correspond to those shown in Figure 2A and B. These sequences were blasted against the human genome via BLAST available at the NCBI to confirm their identity as Kirrel3 splice variants.

**Figure 3:**
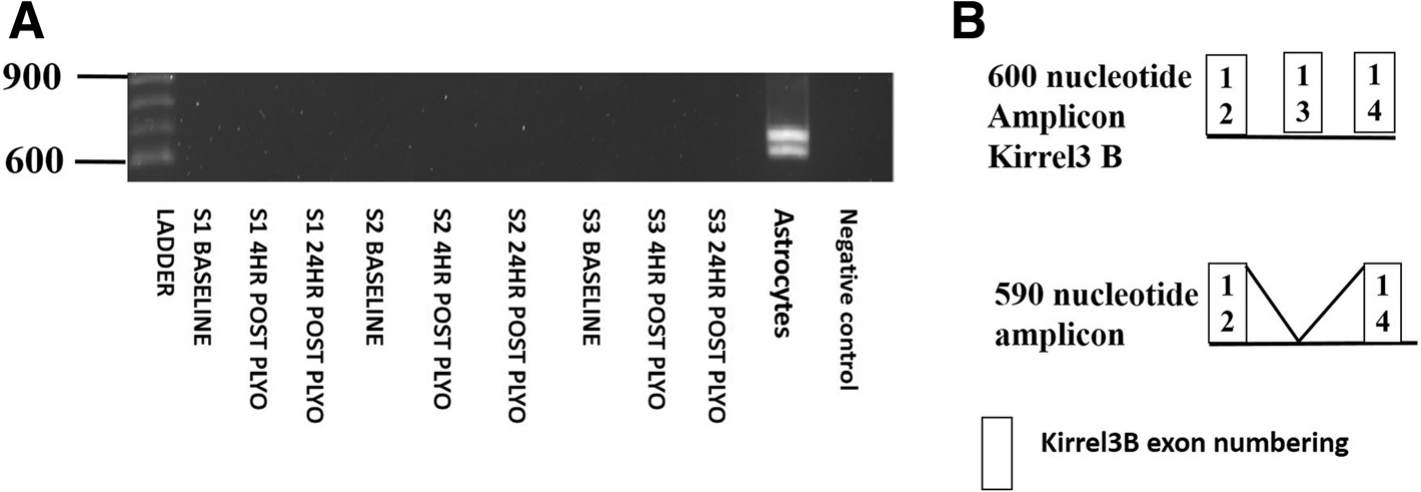
**Detection of Kirrel3 splice variants with primer set 2. A** – PCR amplicons obtained with Kirrel3 primer set 3 that was specific for Kirrel3 B. mRNA was isolated from cultured human astrocytes (2 amplicons present) and adult human skeletal muscle biopsies obtained at rest and 4 and 24 hr post performance of a plyometric jumping exercise (amplicons not evident). **B** – Schematic of the exon structure of both amplicons detected in A based on sequence information.

Subsequently it was of interest to ascertain whether Kirrel3 B mRNA was present in adult human skeletal muscle and astrocytes. The same samples as those from the plyometric jumping exercise intervention were assessed for Kirrel3 B mRNA using primer set 3 that contained the same forward primer as primer set 2 and a reverse primer targeted towards a Kirrel3 B specific sequence present in its 14^th^ exon. The predicted amplicon was 607 nucleotides. No amplicon was detectable in adult human skeletal muscle samples from either the plyometric jumping protocol (Figure 3B) or the downhill running samples (Data not shown), however, two amplicons migrating at approximately 610 and 590 nucleotides were detected in astrocytes (Figure 3B). Both amplicons were excised from the gel and sequenced. The amplicon migrating at approximately 610 nucleotide corresponded to Kirrel3 B while the amplicon that migrated at approximately 590 nucleotides lacked the 13^th^ exon of Kirrel3 B which contains 37 nucleotides (see Table 2 for raw sequence data obtained).

**Table 2:** **Sequence data obtained from amplicons with Kirrel3 primer set 2**

610 nucleotide sequence	AATARMATTGMGAGAGCTATGTGTTCATCCAAAATGCTGGCTGCCCTGCAGATGAAGTTCAGTCTAGTCCAGGTCAACCT
	CAGCCTAGCGCCCACTCTGCCAGGCGCCATGTTGCCCAGGCTCACATACACCGATGCATCAGACCCACTCCCTGCCCCAG
	GAGCTCACAGCACTGTTAGGACCCCTGTTCATTGCACTCCTGCTTACTTGCTCTCCGGGGCAGCCTAAGCCTGGCCTTTT
	TCTCTGTCCCCCTCCCTGAGATCCCGGATCTCCCTCCCGTACTTCTCTGGGAACGGGCACAGCAGAACGCCACGATGGTT
	GCCATAAGGACGAGGAAGGCCACACCAGCTCCTACGGCCACCCCAATGATGACGGCCATCGGCACAGACTCTKGTTCCWK
	GCSSGSTACGAACTTCRTGTCCGARCCTAGSTCCTTGARSGSCATGATSTCGKWGATGGASTGGAAGYTGKYCCRSGCCA
	TGYWGYTGATGATGGKGTGGAAGATGACCCCCTCSATGKTGCTGATGGTCWCGRYCGAAAAGAGRCCCTCCT
590 nucleotide sequence	CATTCGATCGATTRSCGMKAGAGCWTGTGTTCATCCAAATGCTGGCTGCCCTGCAGATGAAGTTCAGTCTAGTCCAGGTC
	AACCTCAGCCTAGCGCCCACTCTGCCAGGCGCCATGTTGCCCAGGCTCACATACACCGATGCATCAGACCCACTCCCTGC
	CCCAGGAGCTCACAGCACTGTTAGGACCCCTGTTCATTGCACTCCTGCTTACTTGCTCTCCGGGGCAGCCTAAGCCTGGC
	CTTTTTCTCTGTCCCCCTCCCTGAGATCCCGGATCTCCCTCCCGTACTTCTCTGGGAACGGGCACAGCAGAACGCCACGA
	TGGTTGCCATAAGGACGAGGAAGGCCACACCAGCTCCTACGGCCACCCCAATGATGACGGCCATCGGCACAGACTCTTGC
	TCCTTGAGCCGGATGATCTCAGTGTCGGAGCCGAAGCTGTTCCAGGCCGTGCAGTTGTAGATGGTCTGGAAGTCGGCCCG
	CACGATGTTGCTGATGGTCAGGGTGGAGATGACGCCCTCCTCGGTGCTGATGGTCTCCACCGTATAGCGA

Amplicons correspond to those shown in Figure 3A. These sequences were blasted against the human genome via BLAST available at the NCBI to confirm their identity as Kirrel3 splice variants.

### Analysis of protein

**Figure 4:**
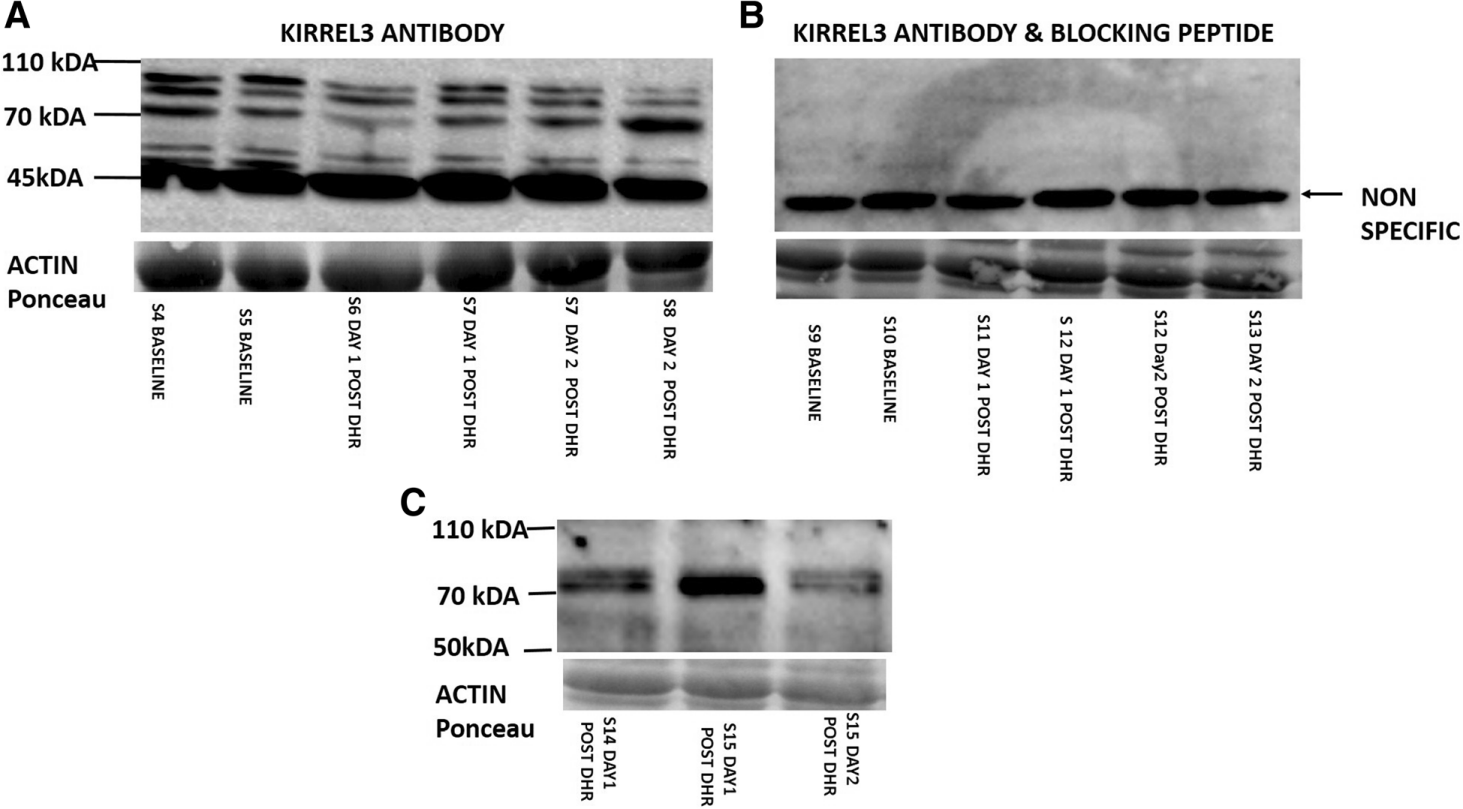
**Kirrel3 protein detection in adult human skeletal muscle. A** – Western Blot results obtained with anti-human Kirrel3 antibody directed toward intracellular region of Kirrel3 on skeletal muscle lysates obtained from adult human males at baseline, days one and two after completion of a DHR exercise protocol. S = subject **B** – Western blot results from blocking peptide experiment. The antibody in A was incubated with blocking peptide before being added to membrane containing skeletal muscle lysates obtained from adult human males at baseline and one and two days post completion of a DHR exercise protocol. **C** – A second commercial antibody directed towards the extracellular domain of Kirrel3 was used on a selection of lysates obtained from adult human male skeletal muscle day1 or 2 post completion of the downhill running exercise bout. Ponceau staining was performed on membranes to demonstrate transfer and equal protein loading.

Based on results obtained from our mRNA analysis we wished to investigate Kirrel3 protein presence in human skeletal muscle samples. A commercially available antibody that was raised against the intracellular AA 596-626 of Kirrel3 A was utilised. Lysates obtained from a previous downhill run (DHR) study by our group [32] were utilised for the analysis. Baseline, 1 and 2 days post DHR were the time points assessed. Multiple immunoreactive proteins were detected in all human skeletal muscle samples ranging in size from approximately 45-110 kDa (Figure 4A). To assess for Kirrel3 specificity of the antibody, a blocking peptide was incubated with the antibody prior to addition to the membrane. The immunoreactive proteins observed at approximately 50-100 kDa with antibody alone were eliminated (Figure 4B), thus providing support that these immunoreactive proteins may be isoforms of Kirrel3. To confirm the protein presence of Kirrel3 a second commercially available antibody that was raised against a recombinant human Kirrel3 protein (AA33-535 of NP_001288026) was tested on a selection of lysates from the downhill running study. Immunoreactive proteins were detected at approximately 70-75 kDA while no immunoreactive proteins were detected at approximately 50-55 kDA and 110 (Figure 4C), which is in contrast to results obtained with the intracellular targeting antibody.

## Discussion

Skeletal muscle is made up of multinucleated cells (muscle fibres). The Drosophila homolog of Kirrel3, Kirre is involved in enabling muscle cell fusion events. Hence, Kirrel3 may be a putative muscle cell fusion regulator in humans. There is currently no published data available regarding Kirrel3 in adult human skeletal muscle. Therefore, the primary aim of this research was to assess for the presence of Kirrel3 in adult human skeletal muscle. By using PCR and transcript sequencing, we provide the first evidence that three alternatively spliced Kirrel3 mRNA transcripts are present in adult human skeletal muscle. Results from western blot analysis provide support for the presence of Kirrel3 protein in adult human skeletal muscle.

The mRNA data highlight that in adult human skeletal muscle Kirrel3 mRNA expression levels are very low and that using standard PCR may result in false negatives. A nested PCR approach yielded a higher rate of Kirrel3 detection. It will be of interest for future studies to examine Kirrel3 mRNA expression in a model that is known to include a large amount of myogenesis such as can be seen in some muscle pathologies. Primary human myoblast cell cultures would provide a useful system to further investigate Kirrel3 and ascertain its importance to human muscle cell fusion. Murine proprioceptive neurons have been reported to express Kirrel3 [33], therefore their contribution to Kirrel3 mRNA expression in adult human skeletal muscle should also be investigated.

The physical process of transcribing Kirrel3 can be regarded as a specialised event because, compared to the vast majority of other genes within the human genome, Kirrel3 spans a very large genomic region of approximately 580kB. Such a long genome span is likely to impact on the rate of Kirrel3 mRNA transcript production. RNA polymerase II has been reported to be capable of transcribing large human genes at a rate of approximately 4 kb^-min^ [34]. This rate of transcription suggests that production of Kirrel3 mRNA transcripts would take approximately 145 minutes. Previously produced transcripts are likely to be translated or degraded within the time period required to produce a Kirrel3 mRNA transcript. Such a time frame may result in a pulsatile occurrence of Kirrel3 mRNA in adult human skeletal muscle, unless Kirrel3 is being continually transcribed. Such a scenario may help explain the apparent discrepancy between the mRNA results in relation to Kirrel3 protein that was detectable in all biopsy samples analysed from both pre and post the DHR exercise intervention. Interestingly, our findings in relation to human Kirrel3 are largely in agreement with two different mouse studies focussing specifically on Kirrel3. In the first study, mRNA has been reported to be absent from mouse skeletal muscle [27], while the second study reported strong protein detection of Kirrel3 in mouse skeletal muscle [28].

Results from our western blot experiments varied depending on the commercial antibody being used. Each antibody targeted different epitopes with one being extracellular and the other intracellular. The fact two immunoreactive proteins that migrated at approximately 70 and 75 kDa were detected by both antibodies, appears to support that these are Kirrel 3 proteins rather than breakdown products. However, the predicted molecular weight of Kirrel3A is 85 kDa therefore the Kirrel proteins within the 70 kDa range are likely to be one or two of the other Kirrel3 splice variants present in human skeletal muscle. It is possible that they represent one splice variant with the larger having undergone post-translational modification. Immunoprecipitation and mass spectrometry analysis will be useful in determining the identity of these immunoreactive proteins.

The proteins detected at 50 and 55 kDa were detected only when using the Kirrel3 antibody targeting the intracellular domain. This antibody may be recognising partially degraded Kirrel3 proteins or these could be alternative truncated isoforms.

For analysis of mRNA, our primers were directed towards the 3′ end region of Kirrel3 (intracellular coding region) and we can only speculate on whether or not alternative splicing may occur further downstream towards the 5′ end (extracellular coding region) that would produce even smaller Kirrel3 protein isoforms. If alternative splicing occurs at the 5′ end this could explain why the extracellular targeting antibody did not detect proteins of similar size as those detected by the intracellular targeting antibody. In future, we suggest that 5′ race experiments should be performed to provide useful information in evaluating this possibility.

Our rationale for investigating Kirrel3 in human skeletal muscle was derived from research studies in Drosophila that demonstrated the involvement of two genes, Kirre and Rst, in the muscle cell fusion process during embryonic development [19]. While our research findings do not prove a role for Kirrel3 in the cell fusion process in adult human muscle, they do raise interesting questions regarding the function of Kirrel3 in human myogenesis and even in uninjured human skeletal muscle fibres. Murine Kirrel3 is present in stromal cells [27] and co-culture studies of Kirrel3 expressing stromal with hematopoietic stem cells, demonstrated a role for the extracellular domain of Kirrel3 in inhibiting hematopoietic stem cells’ differentiation [27]. Such a finding is particularly notable since adult human skeletal muscle also contains stem cells termed satellite cells [14]. Satellite cells, located within the space between the basal lamina and plasma membrane of skeletal muscle fibres, are kept in an undifferentiated state until activated by events such as muscle damage [11]. It will therefore be of future interest to ascertain whether Kirrel3 may be involved in regulating satellite cell differentiation in human skeletal muscle.

In vitro, the extracellular domain of Kirrel3 is capable of binding to the extracellular domain of nephrin, a type 1 transmembrane protein and member of the immunoglobulin superfamily [28]. Nephrin is the mammalian homolog to the Drosophila gene, sticks and stones, that is essential for the muscle cell fusion process during embryonic fly development [20]. Considering that a significant body of evidence has emerged describing similarities in cellular processes between fly and man [35] it is tempting to hypothesise that interaction of Kirrel3 and nephrin in human skeletal muscle may facilitate the muscle cell fusion process. Indeed, in a small mammal model, nephrin^-/-^ murine myoblasts displayed reduced fusion capabilities during in vitro myogenesis [36].

Kirrel3 is part of the Kirrel gene family that contains another two structurally similar members: Kirrel and Kirrel2 [24]. In Drosophila redundancy is present between Kirre and Rst with regard to the muscle cell fusion process [19] and redundancy may also be present among the Kirrel family members for the muscle cell fusion process in humans. Such redundancy should be considered when attempting to ascertain the possible roles of the Kirrel family members in the human muscle cell fusion process. In vitro primary myoblast culture studies should be fruitful in this regard.

The splice variants, Kirrel3 A and B, that are present in adult human skeletal muscle and/or astrocytes are predicted to contain significantly different cytoplasmic domains. Kirrel3 A, but not Kirrel3 B, is predicted to contain a SH3 amphysisin binding domain and also a PDZ binding domain. Such differences are likely to result in the two isoforms having divergent functions. Amphysisin is a protein whose function is not completely understood, however, it is highly concentrated at nerve terminals [37]. The C-elegans homolog of Kirrel3 (an adhesion molecule named syg-1) is located at synapses [38] and hence, amphysisin binding may regulate Kirrel3 A presence at neural synapses or neuromuscular junctions.

The PDZ binding domain present in Kirrel3 A may confer Kirrel3 with the ability to alter the polarity of the cell in which it is expressed as the PDZ binding domain of human Kirrel3 is capable of binding to the cell polarity protein partitioning defective 3 (PARD3) [39]. Satellite cells require PARD3 function in order to achieve asymmetric cell division [40]. The asymmetric cell division of satellite cells enables diversity that is thought to be important in the maintenance of the satellite cell pool. A subset of satellite cells in injured skeletal muscle are highly proliferative while another subset of cells retains the ability to return to quiescence [40]. It will therefore be of future interest to ascertain whether Kirrel3 is present in satellite cells and if so, whether it regulates asymmetric division via its interaction with PARD3.

## Conclusion

In conclusion, the results presented here demonstrate for the first time that there are at least 3 splice variants of Kirrel3 present in adult human skeletal muscle, two of which have never previously been identified in human muscle. Importantly, mRNA of all splice variants was not always present, a finding with potential physiological relevance. These initial discoveries highlight the need for more molecular and functional studies. Full length sequence information should be obtained on all Kirrel3 mRNA transcripts in order to predict the translated Kirrel3 proteins. Physiological studies should be done to confirm involvement in fusion, or not. Production of isoform specific anti Kirrel3 antibodies will enable identification of the cellular localisation of the different Kirrel3 isoforms. Obtaining such information will aid in our understanding of Kirrel3 and its function in human skeletal muscle.

## Methods

### Ethics statement

Healthy young men aged between 18-28 years of age volunteered to participate in one of two studies aiming to describe molecular and physiological responses to exercise-induced muscle damage. Participants were informed about the purpose and risks of the study in which they were to participate before signing an informed consent document. The experimental protocols were approved by the Committee for Human Research at Stellenbosch University and the studies were conducted according to the ethical guidelines and principles of the International Declaration of Helsinki.

### Plyometric jumping protocol

Participants were first taught how to perform the squat jump exercise. Subsequently their maximum squat jump height was measured. For the squat jump exercise intervention participants preformed 100 squat jumps at 90% of their maximum jump height. Jumps were divided into sets of 10 with 1 minute rest interval between each set. Prior to performing the 100 squat jumps participants exercised at a light to moderate intensity for 5 minutes on a treadmill.

### Downhill running protocol

The protocol used here was previously described [32]. In brief, participants performed a 60-minute intermittent DHR protocol (12 × 5 min bouts at 85% VO_2_max, 10% decline) on a motorized treadmill. They were allowed a 2 min standing rest between bouts and all the participants were able to complete all twelve bouts.

### Biopsies

Muscle biopsies were obtained from the *vastus lateralis* muscle using a 5 mm trephine biopsy needle with assisted suction. Baseline biopsies were obtained from participants who had not engaged in any strenuous physical activity 7 days prior to the biopsy. Biopsies from participants who had performed the plyometric jumping protocol were obtained 4 and 24 hrs post completion of the protocol. Baseline and 24 hr biopsies were taken from the right leg while the 4 hr biopsy was taken from the left leg. Similarly, biopsies from participants who completed the downhill running protocol were taken at baseline and one and two days post downhill running in a consistent manner. Biopsies were frozen in liquid nitrogen cooled isopentane and stored at -80°C until use.

### Cell culture

Human astrocytes (SciencCell research laboratories San Diego CA Catalog #1800) were seeded at a density of 10^5^ in 6 well plates (BD Bioscience) in growth media which contained 89% Dulbecco’s Modified Eagle Medium, 1% N-2 Supplement 100X, 10% Fetal Bovine serum (all Life Technologies). Once cells had reached 70% confluence they were lysed for RNA and protein isolation. Growth media was removed from wells before 200 ul of Tripure (Roche) was added per well of a 6 well plate and incubated at room temperature for 5 minutes with occasional gentle agitation. For protein isolation 150 ul of protein lysis buffer was added per well and incubated on ice for 5 minutes with occasional gentle agitation.

### RNA isolation and RT-PCR

Muscle biopsies were sectioned on a cryostat (Leica Bio systems) at -20°C to obtain approximately 20 mg of tissue. Samples were then homogenised on ice in 1 ml of Tripure (Roche). RNA was isolated according to manufacturer’s guidelines and suspended in TE Buffer and stored at -20°C until use. For reverse transcription (RT) 1ug of RNA was DNAse treated according to manufacturers (Roche) guidelines. Subsequently the 1 ug of DNAse treated RNA was reverse transcribed using random hexamers in accordance with manufacturers (Roche – Transcriptor First strand cDNA synthesis kit) guidelines. For PCR a 2 ul aliquot of cDNA corresponding to 50 ng of RNA was used. Each PCR consisted of a total volume of 25 ul. Primers (Sigma Life Science) were used at a 1 uM concentration and remaining components of PCR followed manufacturers (Roche – Faststart PCR Master) recommended protocol. For semi nested PCR a 2 ul volume from the initial PCR was used in the second PCR instead of 2 ul of cDNA. Post PCR the 25 ul volume was mixed with 6 ul of loading buffer (75% v/v glycerol, 0.02% w/V bromophenol blue, 10 mM Tris Base, 1 mM EDTA, 0.2% w/v SDS) and loaded onto a 1% agarose gel which contained sybr safe (Life Technologies) at 1X concentration and electrophoresed alongside a 100 bp ladder (Life Technologies). Gels were visualised on a Chemidoc MP (Biorad) that was supported with Image Lab software (Biorad). Kirrel3 Primer sets used were as follows **Primer set 1 – Forward primer** GCCGACTTCCAGACCATCTA, **Reverse primer –** TTTGAGGACCTCCAGCTGTT, **Primer set 2 - Forward Primer** CGCTATACGGTGGAGACCAT, **Reverse Primer** Same as primer set 1. **Primer set 3 – Forward primer** same as primer set 2, **Reverse Primer –** CGCTTTTCCCCCTATCTTTC. Amplicons were excised from the agarose gel, purified and sequenced at the central analytical facility at Stellenbosch University. For GAPDH primer set used was **Forward primer –** AATCCCATCACCATCTTCCA, **Reverse primer -** TGACAAAGTGGTCGTTGAGG.

### Protein isolation and Western blot

Muscle biopsies were sectioned on a cryostat (Leica Biosystems) at 12 μm to obtain ~20 mg of tissue. Samples were homogenised on ice in 700 ul of lysis buffer (50 mM Tris HCL PH 7.5, 150 mM NaCl, 1 mM EDTA, 1% v/v nonidet p40, 0.25% w/v sodium deoxycholate 1 mM NaF, 1 mM Na3V04, 1 mM PMSF, 1 ug/ml leupeptin, 1 ug/ml pepstatin, 1 ug/ml aprotinin). Protein concentration was measured via a Bicinchoninic acid *(*BCA) kit (Thermo Fischer Scientific) using Bovine serum albumin (Roche) as standards. 30 ug of protein lysate was mixed with 5× Lamelli buffer (60 mM Tris HCL PH6.8, 2% SDS, 10% glycerol, 5% B-mercaptoethanol, 0.01% bromophenol blue) to yield 1× and electrophoresed using the Mini Protean Tetra system (Biorad). Post electrophoresis proteins were transferred to a nitrocellulose membrane (Amsheram Hybond ECL Nitrocellulose membranes GE Healthcare) via Trans Blot Turbo (Biorad) and membranes were stained with ponceau S to confirm transfer and equal protein loading. Subsequently membranes were washed 3 times for 5mins with 1× TBST (50 mM Tris, 150 mM NaCl, 0.1% tween 20, pH 8.3) and then blocked for 1 hr at room temp in 1× TBST and 5% semi skimmed milk. The intracellular targeting primary rabbit anti human Kirrel 3 antibody (102960 Abcam Cambridge UK) and the extracellular targeting primary sheep anti Kirrel 3 antibody (AF4910 R and D systems Minneapolis USA) were incubated over night at room temp with gentle agitation in 1× TBST with 5% semi skimmed milk. For blocking peptide experiment intracellular targeting primary anti human Kirrel 3 antibody was incubated with blocking peptide at a ratio of 1:4 for 3 hrs at room temperature with gentle agitation. Post primary antibody incubation or antibody and blocking incubation membranes were washed 3 times for 5 mins and subsequently incubated either with goat anti rabbit secondary (7074 Cell Signalling) at 1:15,000 or donkey anti sheep HRP conjugated secondary ( 6900 Abcam Cambridge UK) at 1:30,000 for 1 hr at room temp in 1x TBST and 5% milk. Membranes were subsequently washed 6X for 5mins followed by incubation with chemiluminescence detection reagents (Femto, Pierce Thermo Fischer Scientific). Membranes were visualised on a Chemidoc MP (Biorad) which was supported with Image Lab software (Biorad).

## Abbreviations

Kirre: Kin of irregular Chiasm
Rst: Roughest
Kirrel: Kin of irregular chiasm like
Ig: Immunoglobulin
KB: Kilobases
NCBI: National center for biotechnology
AA: Amino acids
PDZ: Post Synaptic density protein 95, Drosophila discs Large tumour suppressor, zonula occludens
